# Work restrictions and Unfitness to work: Prevalence and risk factors a cross-sectional study on 70 000 occupational visits

**DOI:** 10.1371/journal.pone.0353939

**Published:** 2026-07-22

**Authors:** Luther Dogbla, Cédric Gouvenelle, Anne-Sophie Escobar, Florence Thorin, Jiao Jiao, Marek Zak, Julien S. Baker, Bruno Pereira, Frédéric Dutheil

**Affiliations:** 1 Université Clermont Auvergne, CNRS, LaPSCo, Physiological and Psychosocial Stress, CHU Clermont-Ferrand, Occupational Medicine, Clermont-Ferrand, France; 2 Occupational Health service of Cher, Bourges, France; 3 Université Clermont Auvergne, CNRS, Acté, Clermont–Ferrand, France; 4 IDESP, Université Montpellier, INSERM, CHU Montpellier, Montpellier, France; 5 The Jan Kochanowski University of Kielce, Institute of Health Sciences, Collegium Medicum, Kielce, Poland; 6 Hong Kong Baptist University, Sport and Physical Education, Hong Kong; 7 CHU Clermont-Ferrand, Biostatistics, Clinical Research and Innovation Direction, Clermont-Ferrand, France; 8 Université Clermont Auvergne, CNRS, LaPSCo, Physiological and Psychosocial Stress, CHU Clermont-Ferrand, Occupational Medicine, WittyFit, Clermont-Ferrand, France; Federal University of Rio Grande do Sul: Universidade Federal do Rio Grande do Sul, BRAZIL

## Abstract

**Background:**

Few studies have assessed work restrictions and unfitness to work simultaneously, particularly on large populations, and evidence on their main influencing factors remains limited.

**Objective:**

To assess prevalence and risk factors of work restrictions and unfitness to work.

**Method:**

We included all visits conducted in occupational health departments of the Cher, over two consecutive years. Visits had to have a conclusion: fitness to work, temporary work restrictions, permanent work restrictions, and unfitness to work. Mixed multinomial models with individual random effects were used.

**Results:**

Among the 71,848 occupational health visits, 62,436 had a conclusion. Most (90.6%, 95 CI 90.4 to 90.8%) were fit to work, 3.2% (3.1 to 3.3%) had temporary restrictions, 4.9% (4.7 to 5.1%) had permanent restrictions, and 1.3% (1.2 to 1.4%) were unfit to work. The risk of temporary restrictions was multiplied by 1.69 (1.16 to 2.48) in workers over 55 vs < 25 years old; by 1.74 (1.42 to 2.14) in workers with disabilities; by 1.30 (1.07 to 1.58) for workers with 10–20 vs < 5 years of seniority; and by 1.57 (1.22 to 2.01) in companies with 50–199 vs < 10 workers. The risk of permanent restrictions was multiplied by 2.24 (1.68 to 2.97) in workers over 55 yo; by 1.86 (1.57 to 2.21) in workers with disabilities; by 1.47 (1.05 to 2.06) for qualified workers; by 1.24 (1.05 to 1.46) for workers with 10–20 years of seniority; and by 1.30 (1.07 to 1.58) for workers in companies with 50–199 employees. The risk of unfitness to work decreased with increasing seniority in the company, while a significant interaction between sex and sector of activity was observed for temporary restrictions.

**Conclusion:**

Nearly one worker out of ten were unfit to work or had restrictions. The main risk factors for both unfit to work and restrictions were older age, women, and return-to-work visits; whereas lower seniority and smaller companies were risk factors for unfit to work, and longer seniority and bigger companies were risk factors for restrictions.

## Introduction

An occupational health visit is an assessment of an individual’s physical and mental ability to perform the duties of their job [1]. Typically conducted by healthcare professionals such as doctors or nurses, these visits entail physical examinations, medical tests, and reviews of medical histories. The primary aim is to ascertain the individual’s fitness to work and to pinpoint any potential health risks or concerns [2]. Following an occupational health visit, the healthcare professional issues a fitness assessment to work involving specific risks, indicating fitness with or without restrictions. For occupations without particular risks, a follow-up sheet is provided, also with or without restrictions [3]. In cases where the individual is unable to perform their job or their health might be at risk, an unfitness or fitness with restrictions for that specific job is given. Being unfit to work can have a significant impact on an individual’s quality of life and financial stability [4]. It can also lead to a decrease in productivity [5] and put strain on the healthcare system and social welfare programs [6]. Preventing unfitness to work is a key responsibility of occupational health services. There are several actions that individuals and employers can take to prevent or manage unfitness to work [7]. By identifying and managing workers at risk, they can take steps to mitigate the risk of unfitness and prevent it from becoming a permanent condition [8]. However, it’s worth noting that the current body of literature appears to be limited in terms of studies focusing on prevalence of unfit-to-work declaration as well as on characteristics of those workers [9–12] – with few recent publications addressing the factors that contribute to such situations [7,13]. Moreover, those studies focused only on unfit-for-work declaration [9–12], and to our knowledge, no study assessed prevalence of workers with job restrictions. Studying the prevalence of unfit-for-work declaration as well as temporary or permanent restrictions is the basis to build effective preventive strategy, particularly to assess the characteristics of workers the most at risk. Previous research has consistently indicated an increased risk of work unfitness with advancing age [12] but nothing according to restrictions. While some studies found no significant differences between men and women [12], others reported a higher risk of unfitness among women [14,15], and none evaluated a possible interaction between sex and sector of activity [16]. The few studies conducted did not assess the influence of education level on the risk of unfitness-to-work, which is known to be positively linked correlate with physical and mental health [17–19], as well as with access to healthcare [19]. In addition, workers with disabilities represent a particularly vulnerable group regarding work ability and job retention. Previous studies have shown that disability status is associated with increased work limitations, higher risk of work restrictions, and difficulties in maintaining employment [20,21]. To our knowledge, few studies have explored the potential influence of the type of occupational health visit during which unfitness-to-work or work restrictions are issued. Indeed, specific conditions often require particular types of monitoring or adapted visits, that may lead to a declaration of unfitness-to-work [12]. Of course, occupational characteristics of workers can influence the fit-to-work diagnosis. Managers may be more at risk for unfitness, albeit without statistical significant difference [22]. Despite poorly studied, some activity sectors as well as some occupations exhibited a substantially higher risk of unfitness-to-work [9]. However, evidence remains limited regarding the analysis of work restrictions according to occupational characteristics of workers in peer-reviewed studies. An in-depth search only retrieved some reports from occupational health departments or institutions [23,24]. Consequently, there is a knowledge gap in understanding the profiles of workers who may have temporary or permanent restrictions on their fitness for work. Addressing this gap is crucial to gaining a comprehensive understanding of the different dimensions of workforce health, a necessary step to build efficient preventive measures for well-being of workers.

Therefore, the main objective of this study is to ascertain the prevalence of temporary and permanent work restrictions, as well as the prevalence of unfitness for work, while identifying sociodemographic (age, sex, disabilities, education), occupational health (type of visits and type of monitoring), and professional (occupational category, sector of activity, contract, seniority, company size) factors associated with restrictions and unfitness for work.

## Methods

### Study design

The study was approved by the appropriate ethics committee (Comité de Protection des Personnes Sud-Est VI, Clermont-Ferrand, France; N° 2015 / CE 70). This multicentric study retrieved occupational health visits conclusion from eight centers of occupational health, coordinated by the Association de Prévention et de Santé au Travail du Cher (APST18). The APST18 in the Cher operates as part of France’s mandatory occupational health framework, covering approximately 64,000 workers from around 5,700 companies, ensuring health consultations for workers across companies of all sizes and from multiple sectors, distributed as follows: Manufacturing industry (19.5%), Trade, repair of motor vehicles and motorcycles (16.8%), Human health and social work activities (17.3%), Construction (8.4%), Administrative and support service activities (7.0%), Transport and storage (5.2%), Specialized, scientific and technical activities (5.4%), Accommodation and food service activities (4.2%), Financial and insurance activities (4.2%), Other service activities (2.5%), Information and communication (2.8%), Education (1.8%), Water production and distribution (1.8%), and others. Subsidized by employers and the national health system, this service caters to diverse enterprises and is accessible irrespective of company scale. The APST18 covers the follow-up of workers from the specific region of Cher (310,000 inhabitants), in France. We conducted the study over two consecutive years, between January 2021 and December 2022. Data was anonymously extracted from PADOA Software. During visits, workers were informed that anonymized data could be used for research or statistical purposes. No identifying information was collected, and all data were fully anonymized before analysis. Extraction and processing took place between June and August 2023.

### Participants

**Fig 1:**
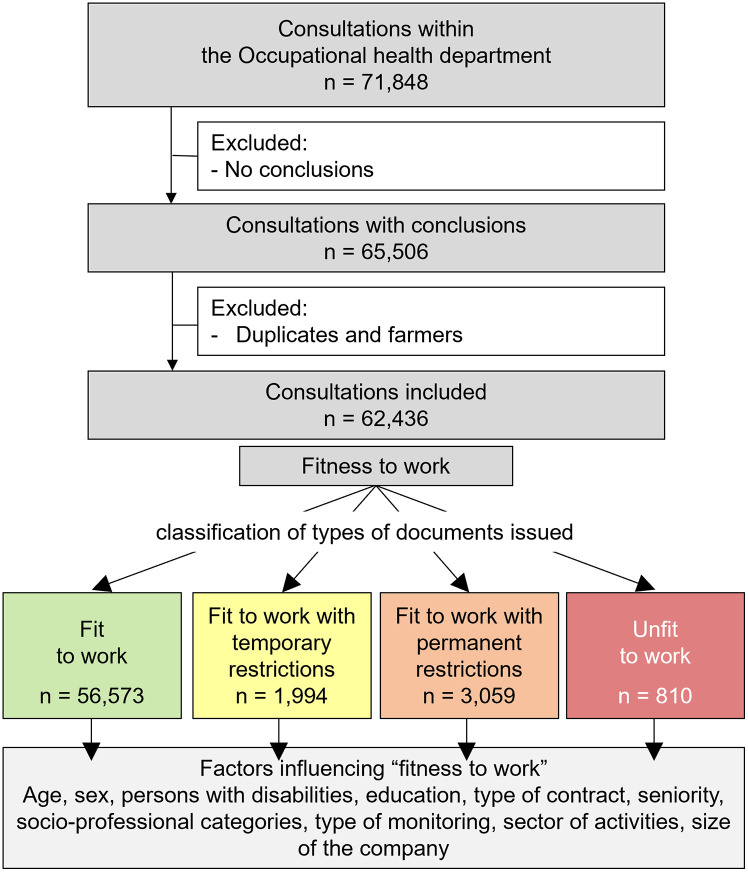
Flow chart.

We included all workers from all sectors except agricultural sector (insufficient data, n = 2 workers). We further included all visits for which an end-of-visit document was issued (Fig 1).

### Main outcome: fitness for work

**Fig 2:**
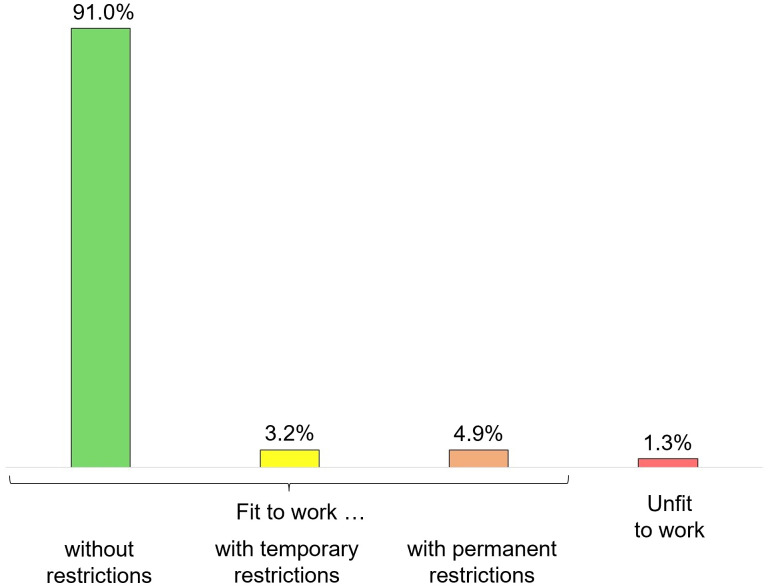
Prevalence of workers depending on fitness for work.

The primary outcome of interest was the determination of fitness for work, categorized into four possibilities: fitness for work, fitness with temporary restrictions, fitness with permanent restrictions, and unfitness for work (Fig 2).

### Secondary outcomes: putative influencing variables

**Fig 3:**
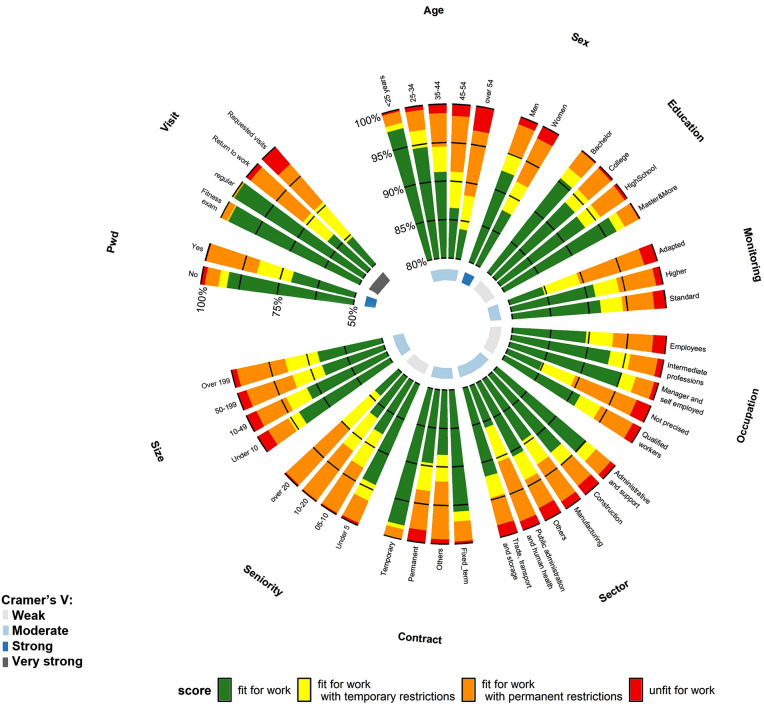
Prevalence of workers by type of fitness for work, depending on sociodemographic, occupational characteristics, and types of visits. Prevalence of type of fitness for work were compared between groups using Cramer’s V. Effect size (ES)>0.05 (weak association); ES > 01.0 (moderate); ES > 0.15 (strong); ES > 0.25 (very strong).

**Fig 4:**
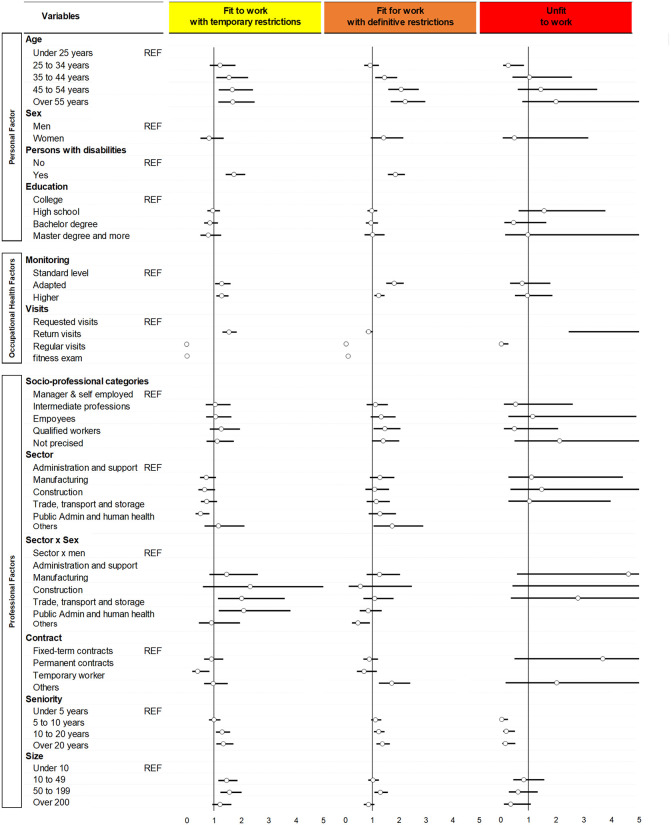
Summary of risk factors for being fit to work with temporary restrictions, fit to work with permanent restrictions, and unfit to work (see details in *Supplementary Figures*). The effect of each variable on the risk of fit to work with temporary restrictions, fit to work with permanent restrictions, or unfit to work is represented by a dot on a horizontal line in the forest-plot. The dots represent the risk (odds ratio) for each variable, and the length of each line around the dots represent their 95% confidence interval (95 CI). The black solid vertical line represents the null estimate (with a value of 1). Odds ratio with horizontal lines that do not cross the vertical line are significant. Significant variables with an odds ratio <1 are protective factors and those with an odds ratio >1 are risk factors. REF: Reference, i.e., the reference for group comparisons.

This study was based on several criteria that have been identified as important in previous research on work-related fitness [12]. We retrieved sociodemographic (age grouped into 10-year increments, sex, disabilities), and occupational health characteristics (type of monitoring for workers, type of visits). Type of monitoring for workers were based on work characteristics and health conditions, in accordance with French occupational health regulations, which define follow-up frequency according to the level of occupational risk and individual worker vulnerability [25]. Type of monitoring for workers were based on work characteristics and health conditions. There are three categories of monitoring: standard (for workers seen every 3–5 years), adapted (for workers seen every 1–3 years), and enhanced (for workers seen every 1–2 years). Four types of occupational health visits were considered: regular medical examinations, regular occupational health visits (Visite d’information et de prévention – VIP), return-to-work visits, and on-demand visits. Medical examinations are conducted to assess the fitness for work of workers. Regular occupational health visits are routine visits for all workers to evaluate their current fitness for work status and to prevent potential health risks. Return-to-work visits are conducted for workers with work-related injuries or illnesses to monitor their recovery and ensure their return-to-work is safe. On-demand visits are conducted at the request of the employee, the employer, or a healthcare professional and are used to evaluate specific health concerns or to conduct targeted assessments. The professional factors that we considered include the type of employment contract (permanent, fixed-term, temporary worker, or other), socio-professional category, the size of the company (grouped by class) and sector of activity (coded according to the European NACE-2008 and international A10 classifications). These standardized classifications allow for comparability across studies and are commonly used in occupational health research. While sector-level categorization can provide insight into patterns of occupational exposure [26] (Figs 3 and 4).

### Statistics

Statistical analysis was performed using Stata® software (v17.0, StataCorp, College Station, TX, US). Categorical data are expressed in number (n), percentage (%) and its associated 95% confidence intervals (95 CI) to provide a measure of precision for the estimates (Table 1). 95 CI were calculated using the tab and CI command in Stata® software. We used Chi-square to compare prevalence of the four possibilities of fitness for work (fitness for work, fitness with temporary restrictions, fitness with permanent restrictions, and unfitness for work) depending on sociodemographic (age, sex, disabilities) and occupational health characteristics (type of monitoring for workers, type of visits). Considering the massive sample size (big data), we also used Cramer’s V for comparisons of prevalence (global overall comparison and two-by-two comparisons). Cramer’s V measures effect size from 0 to 1, where 0 indicates no association, values >0.05 indicate a weak effect, > 0.10 a moderate effect, > 0.15 a strong effect, and >0.25 a very strong relationship [27]. We also presented the results in the form of polar plots to provide a visual representation of the data (Fig 3). In addition to these analyses, we employed a mixed multinomial model with individual random effects to account for variations in the data and to determine the factors associated with the four fitness-to-work possibilities. This approach enhances the robustness of our findings and provides valuable insights into the influence of individual-specific factors. The results were expressed as odds ratios (OR). Forest plots were employed to present the results (Fig 4). We further tested the reliability of our model by comparing our regression analysis to the multinomial logit model without random effect, by using the likelihood ratio test (Chi-square) [28]. We also conducted analyses to assess multicollinearity using the Variance Inflation Factor (VIF). The VIF measures the degree of collinearity among independent variables, and its assessment is vital to ensure the reliability of our regression models [29]. We further performed sensitivity analyses with and without variables containing missing data, as well as conducting analyses with and without individuals who had missing data [30,31]. We also tested potential center effects and the interaction between sex and sectors of activity. A two-sided type I error of 5% was applied for all statistical tests. As such, a difference was considered statistically significant when p < 0.05.

**Table 1:** Prevalence of workers by type of fitness for work, depending on sociodemographic, occupational characteristics, and types of visits. *Prevalence of type of fitness for work were compared between groups using Khi2 and Cramer’s V. Cramer’s V effect size >0.05 are considered weak, > 01.0 moderate, > 0.15 strong, and >0.25 very strong.*

	All	Fit to work…			Unfit to work	P value	Cramer’s V
		Without restrictions	With temporary restrictions	With permanent restrictions			
	n (%)	n (%)	n (%)	n (%)	n (%)		
**Age**							
Under 25 years	10010 (17%)	9758 (97%)	72 (1%)	153 (2%)	27 (0%)	<0.001	0.080 weak
25 to 34 years	13444 (22%)	12714 (95%)	306 (2%)	347 (3%)	77 (1%)		
35 to 44 years	13569 (22%)	12376 (91%)	446 (3%)	589 (4%)	158 (1%)		
45 to 54 years	15473 (24%)	13389 (87%)	727 (5%)	1124 (7%)	233 (2%)		
Over 55 years	9940 (15%)	8336 (84%)	443 (4%)	846 (9%)	315 (3%)		
**Sex**							
Women	26510 (42%)	23302 (88%)	1098 (4%)	1654 (6%)	456 (2%)	<0.001	0.100 Moderate
Men	35926 (58%)	33271 (93%)	896 (2%)	1405 (4%)	354 (1%)		
**Persons with disabilities**							
Yes	2707 (4%)	1934 (71%)	315 (12%)	457 (17%)	1 (0%)	<0.001	0.139 Moderate
No	59723 (96%)	54639 (92%)	1679 (3%)	2602 (4%)	809 (1%)		
**Education**							
College	5065 (10%)	4735 (93%)	121 (2%)	193 (4%)	16 (0%)	<0.001	0.027 Very weak
High school	32684 (62%)	30509 (93%)	785 (2%)	1254 (4%)	136 (0%)		
Bachelor	11071 (22%)	10567 (95%)	192 (2%)	294 (3%)	18 (0%)		
Master’s degree and more	3441 (7%)	3323 (97%)	42 (1%)	71 (2%)	5 (0%)		
**Type of monitoring**							
Standard, every 3–5 years	31922 (52%)	29224 (92%)	917 (3%)	1301 (4%)	480 (2%)	<0.001	0.057 Weak
Adapted, every 1–3 years	7016 (11%)	5944 (85%)	338 (5%)	611 (9%)	123 (2%)		
Higher, every 1–2 years	23498 (38%)	21405 (91%)	739 (3%)	1147 (5%)	207 (1%)		
**Type of visits**							
On-demand visits	4327 (5%)	2871 (66%)	438 (10%)	941 (22%)	77 (2%)	<0.001	0.309 Very strong
Return-to-work	9894 (11%)	6082 (61%)	1469 (15%)	1612 (16%)	731 (7%)		
Regular visits	35914 (63%)	35649 (99%)	41 (0%)	224 (1%)	0 (0%)		
Regular medical examinations	12301 (21%)	11971 (97%)	46 (0%)	282 (2%)	2 (0%)		
**Occupation**							
Manager & self employed	3797 (6%)	3606 (95%)	75 (2%)	94 (2%)	22 (1%)	<0.001	0.048 Very weak
Intermediate professions	9424 (15%)	8764 (93%)	250 (3%)	336 (4%)	74 (1%)		
Employees	18553 (29%)	16626 (90%)	649 (3%)	966 (5%)	312 (2%)		
Qualified workers	25032 (40%)	22802 (91%)	759 (3%)	1181 (5%)	290 (1%)		
Not precised	5630 (8%)	4775 (85%)	261 (5%)	482 (9%)	112 (2%)		
**Sector of activity**							
Manufacturing	13684 (22%)	12248 (90%)	496 (4%)	788 (6%)	152 (1%)	<0.001	0.067 Weak
Construction	4944 (8%)	4594 (93%)	105 (2%)	171 (3%)	74 (1%)		
Trade, transport, and storage	16278 (26%)	14953 (92%)	462 (3%)	602 (4%)	261 (2%)		
Administration and support	9960 (17%)	9476 (95%)	159 (2%)	245 (2%)	80 (1%)		
Public Admin / human health	12933 (20%)	11098 (86%)	613 (5%)	1064 (8%)	158 (1%)		
Others	4637 (7%)	4204 (91%)	159 (3%)	189 (4%)	85 (2%)		
**Contract**							
Permanent contracts	45786 −0.73	41021 (90%)	1665 (4%)	2349 (5%)	751 (2%)	<0.001	0.060 Weak
Fixed-term contracts	5828 (10%)	5585 (96%)	74 (1%)	151 (3%)	18 (0%)		
temporary worker	4079 (7%)	4004 (98%)	21 (1%)	54 (1%)	0 (0%)		
Others	6741 (11%)	5961 (88%)	234 (3%)	505 (7%)	41 (1%)		
**Seniority**							
Under 5 years	32079 (65%)	30239 (94%)	719 (2%)	1010 (3%)	111 (0%)	<0.001	0.084 Weak
5 to 10 years	6858 (13%)	6034 (88%)	344 (5%)	465 (7%)	15 (0%)		
10 to 20 years	6895 (13%)	5971 (87%)	365 (5%)	549 (8%)	10 (0%)		
Over 20 years	5287 (10%)	4446 (84%)	317 (6%)	512 (10%)	12 (0%)		
**Company size**							
Under 10	13965 (23%)	13086 (94%)	228 (2%)	427 (3%)	224 (2%)	<0.001	0.051 Weak
10–49	20883 (34%)	19076 (91%)	620 (3%)	891 (4%)	296 (1%)		
50–199	18355 (29%)	16234 (88%)	747 (4%)	1150 (6%)	224 (1%)		
Over 200	9233 (14%)	8177 (89%)	399 (4%)	591 (6%)	66 (1%)		

## Results

During the study period, 71,848 occupational health visits were recorded, of which 65,506 had conclusions. After removing duplicates, 62,436 visits were included in the analysis. The mean age of workers was 40.8 ± 13.0 years, with men accounting for 42% of visits (Table 1). 4% were with disabilities. Regular visits represented 58% of consultations, and 70% of workers had at least a high school education. Regarding follow-up type, 38% were under enhanced monitoring, 11% under adapted monitoring, and the rest under standard follow-up. Additionally, 63% had less than five years of seniority in their position, and 73% held permanent contracts. Employees made up 30% of the sample, while 40% were qualified workers. The most represented sectors were trade, transport, and storage (26%) and manufacturing (22%). Finally, 33% of visits were in companies with 10–49 workers, and 29% in those with 50–199 workers (Table 1).

### Prevalence of fit-to-work status

Most workers (90.6%, 95 CI 90.4 to 90.8%) were fit for work, 3.2% (3.1 to 3.3%) had temporary restrictions, 4.9% (4.7 to 5.1%) had permanent restrictions, and 1.3% (1.2 to 1.4%) were unfit for work (Fig 1).

### Prevalence of fit-to-work status depending on sociodemographic, occupational health, and professional factors

**Sociodemographic**: Regarding *age*, workers over forty-five years old had the highest prevalence of temporary restrictions (5%), permanent restrictions (8%), as well as unfitness for work (2%) (vs 1%, 2% and 0% for those under twenty-five years old, respectively; Cramer’s V = 0.080, i.e., a weak association). Regarding *sex*, women had a higher prevalence of temporary restrictions (4%), permanent restrictions (6%), and unfitness for work (2%) (vs 2%, 4% and 1% for men, respectively; Cramer’s V = 0.100, i.e., a weak association). Regarding *disability* status, workers with disabilities had a higher prevalence of temporary restrictions (12%) and permanent restrictions (17%) compared with workers without disabilities (3% and 4%, respectively) (Cramer’s V = 0.139, i.e., a moderate association).

**Occupational health**: Considering *type of monitoring*, workers with an adapted monitoring had the highest prevalence of both temporary (5%) and permanence (9%) restrictions (vs 3% and 4% for standard and enhanced monitoring, Cramer’s V = 0.057, i.e., a weak association). Considering *type of visits*, return-to-work visits and on-demand visits had the highest prevalence of temporary restrictions (13%), permanent restrictions (18%), and unfitness for work (6%) (vs 0%, 1%, and 0% for regular visits and regular medical examinations; Cramer’s V = 0.309, i.e., a very strong association).

**Professional factors**: For *occupational categories*, managers and self-employed workers had the lowest prevalence (2%) of both temporary restrictions and permanent restrictions (vs 3 and 5% for other occupational categories, respectively). 2% of employees were unfit for work (vs 1% for other categories, Cramer’s V = 0.048, i.e., a very weak association). In terms of *sector of activity*, public administration/human health and manufacturing had the highest prevalence of temporary (4%) and permanent (7%) restrictions (compared with 2% and 3% for other sectors), whereas unfitness for work was the most prevalent in the trade, transport, and storage sector (2%) (Cramer’s V = 0.067, i.e., a weak association). By *type of contract*, workers with a permanent contract had systematically the highest prevalence of temporary restrictions (4%), permanent restrictions (5%), and unfitness for work (2%) (compared with 1%, 1%, and 0% for temporary workers; Cramer’s V = 0.060, i.e., a weak association). Workers with over 20 years of *seniority* had the highest prevalence of temporary (6%) and permanent (10%) restrictions (vs 2 and 3% for workers with less than five years of seniority, respectively; Cramer’s V = 0.084, indicating a weak association) – without difference for unfitness for work. For *company size*, larger companies over fifty workers had the highest prevalence of temporary (4%) and permanent (4%) restrictions (vs 2 and 3% for small companies with less than ten workers, respectively), whereas the highest prevalence of unfitness for work was opposite, i.e., 2% for small companies vs 1% for large companies (Cramer’s V = 0.051, i.e., a weak association) (Table 1, Fig 3).

### Fitness status risk factors (odds ratio)

The mixed multinomial model with individual-level random effects was significantly superior to the classical multinomial logit model in explaining our dataset (chi2 = 48.5, p < 0.001). In addition, the inclusion of the sex × sector interaction significantly improved model fit (chi2 = 36.9, p = 0.0013).

**Sociodemographic**: There is a dose response relation between age and the risk of restrictions. Compared to those under 25 years old, the risk of temporary restrictions was multiplied by 1.56 (95% CI 1.10 to 2.25) for workers aged 35–44 years old (yo), by 1.68 (1.17 to 2.60) for those aged 45–54 yo and by 1.69 (1.16 to 2.48) over 55 yo. The risk of permanent restrictions was multiplied by 1.46 (1.11 to 1.93) for the 35–44 yo workers, by 2.09 (1.59 to 2.74) for those 45–54 yo, and by 2.24 (1.68 to 2.97) over 55 yo. There is a similar pattern, i.e., a gradual increasing risk of unfitness for work with age: a risk multiplied by 0.28 (0.09 to 0.84) for the 25–34 yo workers, by 1.05 (0.43 to 2.57) for 35–44 yo, by 1.46 (0.61 to 3.49) for those 45–54 yo, and by 2.00 (0.78 to 6.90) over 55 yo. Being a woman was not associated with temporary restrictions (0.82, 0.50 to 1.36) but tended to increase the risk of permanent restrictions (1.42, 0.94 to 2.15) and was not associated with unfitness for work (0.49, 0.08 to 4.90). There was no effect of education level on fitness status, whereas disabilities were associated with a higher risk of both temporary (1.74, 1.42 to 2.14) and permanent (1.86, 1.57 to 2.21) restrictions.

**Occupational health**: Compared with a standard *monitoring*, both an adapted and an enhanced monitoring multiplied the risk of temporary restrictions by 1.29 (1.03 to 1.61) and 1.29 (1.09 to 1.54), and the risk of permanent restrictions by 1.82 (1.52 to 2.17) and 1.25 (1.08 to 1.46), respectively. The type of monitoring did not influence the risk of unfitness. Compared with requested visits, return-to-work visits multiplied the risk by 1.56 (0.50 to 1.83) for temporary restrictions and by 0.86 (0.75 to 1.01) for permanent restrictions; regular visits were associated with a markedly lower risk (0.01, 0.00 to 0.01 and 0.02, 0.02 to 0.03, respectively); and fitness examinations were also associated with a lower risk (0.03, 0.01 to 0.04 and 0.11,0.09 to 0.13), respectively). Regarding unfitness for work, requested visits showed a strongly increased risk (6.29, 2.45 to 16.11), whereas regular visits (0.03, 0.00 to 0.29) and fitness examinations were associated with a lower risk; no estimate could be derived for return-to-work visits (Fig 4).

**Professional factors**: Qualified workers had a 47% increased risk of permanent restrictions (1.47, 1.05 to 2.06) compared to managers. No clear sector effect was observed for permanent restrictions, except for the “Other sectors” category (1.74, 1.05 to 2.91), while sector was not associated with temporary restrictions. For unfitness for work, no sector reached statistical significance. Interaction Sex x Sector of activity showed that women had an increased risk of temporary restrictions compared to men, by 102% (2.02, 1.14 to 3.58) when working in the Trade, transport and storage, and by 110% (2.10, 1.17 to 3.79) in Public administration and human health. Conversely, women working in Other sectors had a lower risk of permanent restrictions (0.47, 0.25 to 0.91). In comparison with small companies (<10 workers), the risk of temporary restrictions was multiplied by 1.46 (1.16 to 1.86) for companies with 10–49 workers, and by 1.57 (1.22 to 2.01) for those with 50–199 workers. The risk of permanent restrictions was multiplied by 1.30 (1.07 to 1.58) in 50–199 companies. There seem to be a dose response decrease in the risk of unfitness for work with bigger size of companies (0.84, 0.45 to 1.57 for 10–49; 0.63, 0.30 to 1.34 for 50–199; 0.37, 0.12 to 1.10 for ≥200 workers). Similarly, there is a dose response effect between restrictions and seniority. The risk of temporary restrictions was multiplied by 1.00 (0.82 to 1.23) for 5–10 years of seniority, by 1.30 (1.07 to 1.58) for 10–20 years, and by 1.36 (1.10 to 1.71) for over 20 years. 1.13 (0.95 to 1.33), 1.24 (1.05 to 1.46), and 1.38 (1.16 to 1.66), respectively. In contrast, the risk of being unfit for work decreased with increasing seniority (0.04, 0.00 to 0.27; 0.21, 0.09 to 0.51; and 0.18, 0.06 to 0.53 for 5–10, 10–20 and >20 years, respectively). Contract type did not exhibit any statistically significant effect across fitness status (Fig 4).

No significant center effect was observed, indicating that the results were consistent across the participating centers.

## Discussion

The main findings were that most workers (90.6%) were fit for work, 3.2% had temporary restrictions, 4.9% permanent restrictions, and 1.3% were unfit for work. Main risk factors for work restrictions or unfitness were older age and women (sociodemographic); return visits and higher monitoring (occupational health); and opposite effects for seniority and company size – higher seniority and larger company size being protective for unfitness but a risk for restrictions (professional factors).

### Prevalence of restrictions and unfitness for work

The high rate of fitness for work is encouraging and may reflect the effectiveness of prevention policies and company health promotion programs. However, we found a notably higher prevalence of unfitness for work (1.3%) than the 0.7% reported in other studies [12,14,15]. Several factors may contribute to this discrepancy. Most studies considered permanent unfitness for work, which may underrepresent the complexity and true prevalence of work-related unfitness in their populations [14]. However, the classification system is more complex and distinguishes between different types of unfitness: unfitness for all jobs, unfitness for specific jobs, and unfitness without reclassification. In our study, our category of unfitness for work comprises all aforementioned type of unfitness, which may explain difference with prevalence reported in the literature. Interestingly, to our knowledge, there are no studies that specifically reported the prevalence of work restrictions, either temporary or permanent, highlighting the novelty of our study. Work restrictions is very important as they can be considered as a preventive strategy to decrease the risk of unfitness for work [7,8]. Longitudinal studies are necessary to assess the effects of proactive management on fitness for work. Unfitness for work may be linked to serious conditions or to unsuitable working environments [15,32]. Studies showed that unfitness for work has significant economic consequences for both individuals and companies, including reduced productivity [4–6]. As many workers are fit, efforts can focus on supporting workers with restrictions and on initiatives to prevent unfitness for work.

### Sociodemographic and fitness status

The higher likelihood of work restrictions with older age can be attributed to age-related declines in physical and cognitive functioning [33–36]. Older individuals are also more susceptible to age-related health conditions, further affecting their fitness for work [37]. Differences in work restrictions and unfitness between men and women stem from several factors, including anatomical differences like physical strength and body size [16,38,39], and the uneven distribution of occupational roles and exposures [38,39]. Social interactions and family obligations also play a role, impacting working conditions and occupational risks differently for men and women [40,41]. Interestingly, we showed that women were more at risk of temporary restrictions than men, for those working in the trade, transport and storage, as well as in public administration and human health, which is coherent with possible exposure to physical and psychosocial risk factors in those sectors, respectively women being likely more susceptible to those risk factors [16,38,39]. We also observed that workers identified as persons with disabilities had a markedly higher likelihood of both temporary and permanent work restrictions, which is consistent with the presence of underlying health limitations and with occupational health policies aiming to maintain employment through job adaptation rather than exclusion from work [21]. Our study did not find a significant impact of education level on the risk of unfitness for work, although previous research has shown that higher educational attainment is often linked to better physical and mental health outcomes [17,18]. One possible explanation for this discrepancy is that educational level may have an indirect protective effect by facilitating access to less physically demanding jobs, more autonomy at work, or greater adaptability to job changes factors not fully captured in our analysis. Moreover, the relatively limited variability in education levels within our sample could have reduced the power to detect such associations. Higher education may also support job retention among workers who might otherwise be deemed unfit for any position in the company [42,43]. Further research is needed to explore these mechanisms and their contextual specificities.

### Occupational health and fitness status

In our study, the diverse types of occupational health monitoring — reinforced, adapted, or standard — significantly influenced conclusions about work fitness and restrictions. Workers under adapted monitoring were more likely to have permanent restrictions due to the need for job adaptations to achieve optimal productivity among workers with disabilities [44,45]. However, no disparity in unfitness risk was observed between these workers and others, suggesting the adequacy of the monitoring approach in addressing varying exposure levels and worker pathologies [46]. Return-to-work visits, which showed the highest prevalence of unfitness, are scheduled after at least 60 days of absence in France – and 30 days after an absence for a work-related accident or an occupational disease, according to Article R4624-31 of the French Labor Code. Despite recognition by occupational health professionals, no study has extensively demonstrated the higher prevalence of unfitness risk or increased restrictions during return-to-work visits. This underscores the increased risk of incapacity or job restrictions with significant health changes on-demand visits also highlight issues by providing a platform for workers, employers, and healthcare professionals to report health status and workplace condition alerts [47].

### Professional factors and fitness status

In our study, qualified workers exhibited the highest rates of unfitness and restrictions, likely due to significant biomechanical constraints in their roles, unlike managerial positions [36,48,49]. This highlights the importance of ergonomics and occupational risk prevention in physically demanding environments [50]. In line with our results, construction and commerce sectors showed higher unfitness due to physically demanding tasks and hazardous conditions, while public administration and healthcare workers face psychosocial stress and long hours, leading to restrictions [51–54]. Strict regulations in these sectors can limit flexibility in accommodating workers with health conditions [55]. We also observed a significant interaction between sex and sector of activity: women had a higher likelihood of temporary work restrictions than men in the trade, transport and storage, and in public administration and human health. This may reflect gendered task distribution and differential exposure to physical and psychosocial constraints within these sectors [41]. In our study, workers with permanent contracts surprisingly showed higher rates of work restrictions and unfitness, contrary to what might be expected. Although this effect has not been extensively explored in the literature, we find that fixed-term contracts contribute to higher stress and adverse health effects, impacting fitness for work, with workers having less control over their environment [56–58]. Previous studies showed that, those workers may experience reduced job security, limited benefits, and fewer opportunities for training and development compared to permanent workers [59]. Although the Danish flexicurity system offers some job security, it doesn’t justify specific interventions, as contract effects on health vary [57]. In our findings, shorter seniority was associated with a higher risk of unfitness to work, whereas longer seniority was linked to more frequent work restrictions. This may be explained by the fact that seniority increases restrictions due to prolonged exposure to risks, though senior workers often show greater job attachment [42,60]. Small companies have higher unfitness rates and fewer restrictions due to limited resources and less formal management [61–63]. In contrast, large companies, with robust health and safety policies, better manage unfitness and offer more reassignment options, partly due to legal obligations to accommodate disabled workers [45,64].

### Limitations

While this study provides valuable insights into occupational health visits, several limitations should be acknowledged. Firstly, the retrospective design may introduce selection bias [65], as visits were based on available records rather than a predefined sampling method. Additionally, the study focused on visits with issued documents, and, despite uncommon, we cannot exclude that some visits were conducted but not documented [66]. Moreover, although our study is not based on national data, it includes a large dataset from multiple occupational health centers within the Cher region, which strengthens the representativeness of our findings at the regional level. It should be noted that occupational health legislation and evaluation practices differ across countries, limiting direct comparability. Furthermore, the absence of data on certain variables, such as specific health conditions or work-related factors, safety measures in companies, may restrict the depth of analysis. Additionally, detailed medical diagnoses or ICD codes were not consistently available in the dataset, which limits the ability to link specific medical conditions to work restrictions or unfitness. Lastly, the observational nature of the study precludes causal inference, and unmeasured confounders may influence the observed associations [67]. Despite these limitations, this study contributes valuable insights into occupational health visits and provides a foundation for further research in this area. Moreover, beyond the factors identified in our study, the assessment of fitness for work remains a complex clinical process that relies on a comprehensive evaluation of job tasks, occupational exposures, and work environment, combined with medical history, physical examination, and complementary tests; it also highlights the importance of adequate training of occupational health professionals to ensure accurate and consistent evaluations.

## Conclusion

Most occupational visits of workers (>90%) had a fit for work diagnosis, 3.2% had temporary restrictions, 4.9% had permanent restrictions, and 1.3% (1.2 to 1.4%) were unfit for work. The risk of restrictions or unfitness for work increased in older workers, women, those seen in occupational health for a return visit, and workers with a high seniority – interestingly, small companies exhibited more unfitness cases, while larger companies had more restrictions. Future research could explore the role of specific medical conditions, job demands, and international comparisons to further refine preventive strategies for work restrictions and unfitness.

## Supporting information

S1 File Risk factors for being fit to work with temporary restrictions, fit to work with permanent restrictions, and unfit to work. The effect of each variable on the risk of fit to work with temporary restrictions, fit to work with permanent restrictions, or unfit to work is represented by a dot on a horizontal line in the forest-plot. The dots represent the risk (odds ratio) for each variable, and the length of each line around the dots represent their 95% confidence interval (95 CI). The black solid vertical line represents the null estimate (with a value of 1). Odds ratio with horizontal lines that do not cross the vertical line are significant. Significant variables with an odds ratio <1 are protective factors and those with an odds ratio >1 are risk factors. REF: Reference, i.e., the reference for group comparisons.
(TIF)
