# Supplementary figures and images for: Work restrictions and Unfitness to work: Prevalence and risk factors a cross-sectional study on 70 000 occupational visits
Source: PLoS One. 2026 Jul 22;21(7):e0353939. doi: 10.1371/journal.pone.0353939 (PMC13390862; doi:10.1371/journal.pone.0353939)

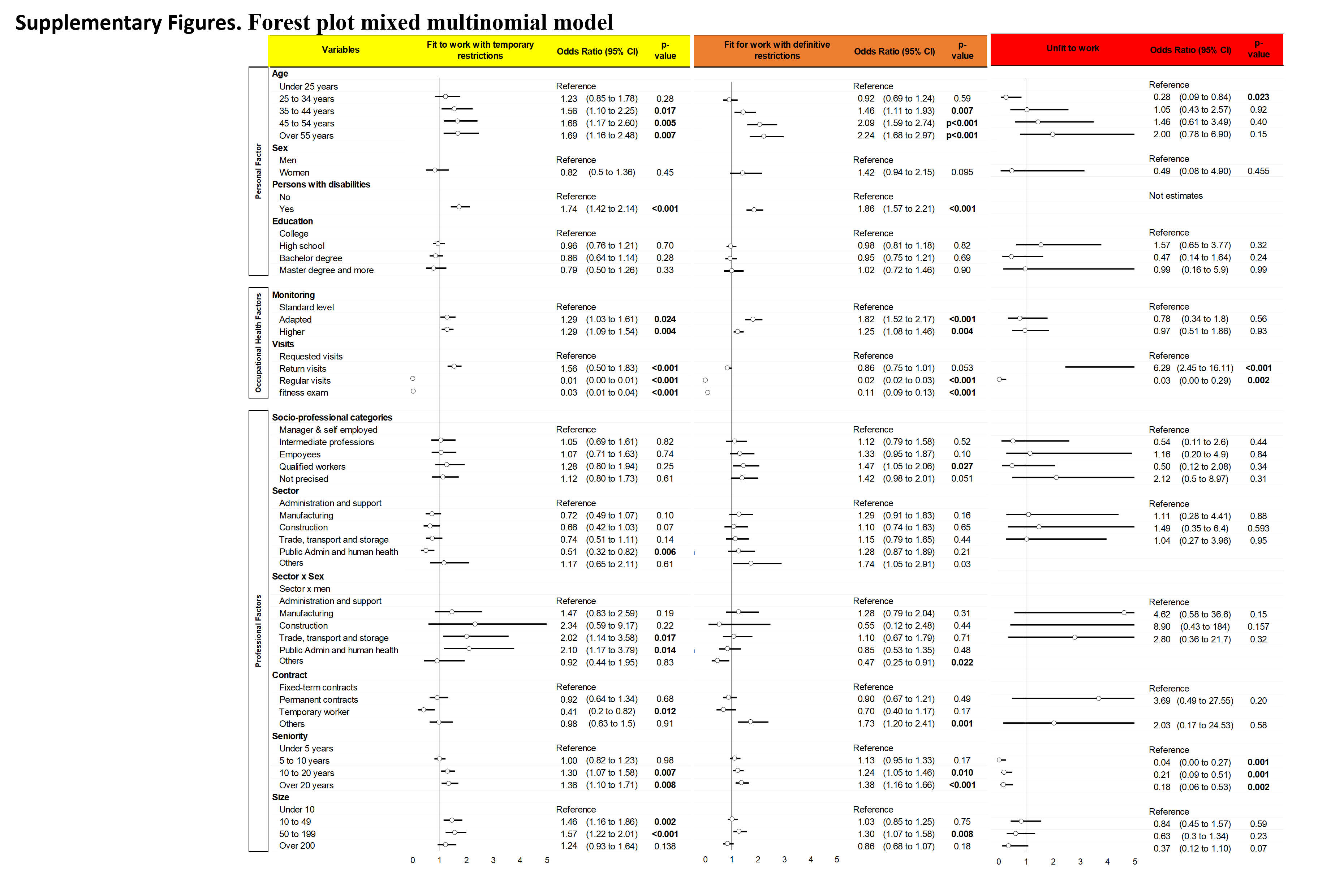

Supplement: S1 File — The effect of each variable on the risk of fit to work with temporary restrictions, fit to work with permanent restrictions, or unfit to work is represented by a dot on a horizontal line in the forest-plot. The dots represent the risk (odds ratio) for each variable, and the length of each line around the dots represent their 95% confidence interval (95 CI). The black solid vertical line represents the null estimate (with a value of 1). Odds ratio with horizontal lines that do not cross the vertical line are significant. Significant variables with an odds ratio <1 are protective factors and those with an odds ratio >1 are risk factors. REF: Reference, i.e., the reference for group comparisons. (TIF) [file pone.0353939.s001.tif]
